# Reviewer acknowledgement 2013

**DOI:** 10.1186/1749-8090-9-26

**Published:** 2014-01-31

**Authors:** Vipin Zamvar, David Taggart

**Affiliations:** 1 Royal Infirmary of Edinburgh, Edinburgh, UK; 2 John Radcliffe Hospital, Oxford, UK

## Abstract

**Contributing reviewers:**

The Editors of *Journal of Cardiothoracic Surgery* would like to thank all our reviewers who have contributed to the journal in Volume 8 (2013).

## 

Fuad Abdullayev

Azerbaijan

Razi Abuanzeh

United Kingdom

Tarek Abulezz

Egypt

Fabio Acocella

Italy

Vera Aiello

Brazil

Clemens Aigner

Austria

George Aislaitner

Greece

Atif Akcevin

Turkey

Ibrahim Akin

Germany

AbdulRahman AlBlooshi

United Arab Emirates

Luciano Albuquerque

Brazil

Ivan Aleksic

Germany

Jorge Almeida

Portugal

Rui Almeida

Brazil

Faisal Al-Mufarrej

United States of America

Bahaaldin Alsoufi

Saudi Arabia

Peter Alston

United Kingdom

Peter Alston

United Kingdom

Mohammad Al-Tarshihi

Jordan

Miguel Álvarez

Spain

Khalid Amer

United Kingdom

Antonio Amodeo

Italy

Yasser Amr

Egypt

Qi An

China

Dimitrios Angouras

Greece

Francisco Arabia

United States of America

Mihalis Argiriou

Greece

Tohru Asai

Japan

Boulos Asfour

Germany

Fatih Asgun

Turkey

Harun Avdagic

Bosnia and Herzegovina

Koray Aykut

Turkey

Dalim Kumar Baidya

India

Richard Baillot

Canada

Enzo Ballotta

Italy

Nikolaos Baltayiannis

Greece

David Balzer

United States of America

Richard Bambury

United States of America

Paul Barach

Australia

Sion Barnard

United Kingdom

Michael Bates

United States of America

Ceri Battle

United Kingdom

Hans Joerg Baumann

Germany

Jeroen J. Bax

Netherlands

Wilhelm Behringer

United Arab Emirates

Edward Bender

United States of America

Umberto Benedetto

United Kingdom

Vincent Benouaich

France

Stefano Benussi

Italy

Geoffrey Berg

United Kingdom

Jacob Bergsland

Norway

Olivier Bertrand

Canada

Farah Bhatti

United Kingdom

Brian Biggers

United States of America

Chantal Bleeker-Rovers

Netherlands

Nikolaos Bonaros

Austria

Johannes Bonatti

United Arab Emirates

Raoul Borioni

Italy

Wolfgang Bothe

Germany

Mohammed Bouchikh

Morocco

Carlos Brandao

Brazil

Norman Briffa

United Kingdom

Katherine Brown

United Kingdom

Harold Burkhart

United States of America

Antonio Calafiore

Saudi Arabia

Emilio Canalis

Spain

Luiz Fernando Caneo

Brazil

Gaetano Caramori

Italy

Giuseppe Cardillo

Italy

Filip Casselman

Belgium

Nicholas Cavarocchi

United States of America

Jose Ceron

Spain

Hakan Ceyran

Turkey

Suchart Chaiyaroj

Thailand

David Chambers

United Kingdom

Guangqi Chang

China

Didier Chatel

France

Caroline Cheng

Netherlands

Cai Cheng

China

Bruno Chiappini

Italy

Jong BUm Choi

South Korea

Namsik Chung

South Korea

Tomás Cianciulli

Argentina

Mustafa Cikirikcioglu

Switzerland

Paola Ciriaco

Italy

John Clark

United States of America

Massimo Conti

Canada

Monica Contino

Italy

Charles Cook

United States of America

Francisco Costa

Brazil

Carlos Costa Almeida

Portugal

Yazhou Cui

China

Suzanne Dahlberg

United States of America

Andrea Daprati

Italy

Ahmad Darwazah

Israel

Jan De Raet

Germany

Mircea Dediu

Romania

Eva Maria Delmo-Walter

Germany

Hayati Deniz

Turkey

Peter Denk

United States of America

Rogier Determann

Netherlands

Roberto Di Bartolomeo

Italy

Robert Di Domenico

United States of America

Salvatore Di Stefano

Spain

Luigi Di Tommaso

Italy

Li-Wei Diao

China

David Dichek

United States of America

Vassilios Didilis

Greece

Fabien Doguet

France

Carlos Donayre

United States of America

Dimitrios Dougenis

Greece

Daniel John Doyle

United States of America

Mats Dreifaldt

Sweden

Xiao-Jun Du

Australia

Christian Duale

France

Joel Dunning

United Kingdom

Tjark Ebels

Netherlands

Sonia Eiras

Spain

Amir Elami

Israel

Bilgin Emrecan

Turkey

Juergen Ennker

Germany

Leonardo Esteves Lima

Brazil

Anthony Estrera

United States of America

Paulo Roberto Evora

Brazil

Olivier Facy

France

Bode Falase

Nigeria

Daping Fan

United States of America

Jun Fang

China

Khalil Fattouch

Italy

Levente Fazekas

Hungary

Quan Zhou Feng

China

Enrico Ferrari

Switzerland

Marcelo Ferreira

Brazil

Richard Feyrer

Germany

Vincent Figueredo

United States of America

Gokturk Findik

Turkey

Juergen H. Fischer

Germany

P. Marco Fisichella

United States of America

Tatjana Fleck

Austria

Christophoros Foroulis

Greece

Alain Fraisse

France

Richard Freeman

United States of America

Sara Fuentes

Spain

Toshihiro Fukui

Japan

Rafael García Fuster

Spain

Rocío García Orta

Spain

Avihu Gazit

United States of America

Guido Gelpi

Italy

Michele Genoni

Switzerland

James George

United States of America

Shane George

United Kingdom

Suat Gezer

Turkey

Kari Hanne Gjeilo

Norway

Onur Goksel

Turkey

Hanna Golab

Netherlands

Steven Goldberg

United States of America

Renata Gomes

Portugal

Walter Gomes

Brazil

Susan Goobie

United States of America

Masashi Gotoh

Japan

Antonino Grande

Italy

Hans Granfeldt

Sweden

Adams Grant

Germany

S Bruce Greenberg

United States of America

Michael Grimm

Austria

Elissavet Grouzi

Greece

Neil Grubb

United Kingdom

Tianxiang Gu

China

Murat Guclu

Saudi Arabia

Helmut Gulbins

Germany

Adem Guler

Turkey

Serdar Gunaydin

Turkey

Changfa Guo

China

Junming Guo

China

Seok Jin Haam

South Korea

Rune Haaverstad

Norway

Majid Haghjoo

Iran

Shuji Haraguchi

Japan

Syed Faisal Hashmi

United Kingdom

Jianxing He

China

Andre Hebra

United States of America

Khosro Hekmat

Germany

Michael Hess

United States of America

Yasuhiro Hida

Japan

Adam Hill

United Kingdom

David Hoganson

United States of America

Jürgen Hörer

Germany

Mitsuharu Hosono

Japan

Xiaotong Hou

China

Han-Shui Hsu

Taiwan

Ron-Bin Hsu

Taiwan

Charles Huddleston

United States of America

Dongmyung Huh

South Korea

Joseph Huh

United States of America

Gianluca Iacobellis

United States of America

Junsuke Igarashi

Japan

Andrea Imperatori

Italy

Kei Inai

Japan

Hiroshi Ito

Japan

Yasuhiro Ito

Japan

Marjan Jahangiri

United Kingdom

Eric Jamieson

Canada

Robert Jaquiss

United States of America

kalyana javangula

United Kingdom

Olivier Jegaden

France

Hongwen Ji

China

Qiang Ji

China

Gening Jiang

China

Mark Jones

United Kingdom

Rajnish Joshi

India

Lyle Joyce

United States of America

Christian Jung

Germany

Tae-Eun Jung

South Korea

Huseyin Kadioglu

Turkey

Ashwini Kalantri

India

Ayeesha Kamran Kamal

Pakistan

Yukihiro Kaneko

Japan

Emmanouil Ioannis Kapetanakis

Greece

Mehmet Kaplan

Turkey

Melih Kaptanoglu

Turkey

Evvah Karakilic

Turkey

Dimitrios Karantanis

Greece

Eren Karpuzoglu

Turkey

Iraklis Katsoulis

Greece

Pankaj Kaul

United Kingdom

Toshiaki Kawai

Japan

Teruhisa Kazui

Japan

Arndt Kiessling

Germany

Gun Jik Kim

South Korea

Hong Kwan Kim

South Korea

Ki-Bong Kim

South Korea

Se-Chan Kim

Germany

Yongin Luke Kim

South Korea

Young Tae Kim

South Korea

Sohei Kitazawa

Japan

Uwe Klima Klima

United Arab Emirates

Chris Klonaris

Greece

Christopher Knott-Craig

United States of America

Toshiro Kobayashi

Japan

Bülent Koçer

Turkey

Alfred Kocher

Austria

Alexander Kogan

Israel

Takushi Kohmoto

United States of America

Hakan Köksal

Turkey

Efstratios Koletsis

Greece

Steffen Kolschmann

Germany

Tatsuhiko Komiya

Japan

Kemal Korkmaz

Turkey

Grigorios Korosoglou

Germany

Dimitrios Koudoumas

United States of America

Thomas Krasemann

United Kingdom

Rafal Krenke

Poland

Sreedhar Krishna

United Kingdom

Tobias Kruger

Germany

Hiroshi Kubota

Japan

Manoj Kuduvalli

United Kingdom

Ferdinand Kuhn-Regnier

Germany

Sanath Kumar

United States of America

TK Susheel Kumar

United States of America

Chih-Hsi Kuo

Taiwan

Mariusz Kusmierczyk

Poland

Jean-Pierre Laaban

France

Peter Lamb

United Kingdom

Michael Lanuti

United States of America

Rifat Latifi

United States of America

Omar Lattouf

United States of America

Mark Law

United States of America

Incilay Lay

Turkey

João Carlos Leal

Brazil

DongHyup Lee

South Korea

Sub Lee

South Korea

Christian LeGuern

United States of America

Tiago Leiria

Brazil

Massimo Lemma

Italy

Xue-Feng Leng

China

Jerrold Levy

United States of America

Weiguo Li

United States of America

Stefan Limmer

Germany

Luiz Lisboa

Brazil

KeXiang Liu

China

Kun Liu

China

Antonio Loforte

Italy

Hao Long

China

Gabriel Loor

United States of America

Juan Carlos Lopez-Delgado

Spain

Roberto Lorusso

Italy

Mahmoud Loubani

United Kingdom

Marco Lucchi

Italy

Justin Lundbye

United States of America

Kevin Maher

United States of America

Zeena Makhija

India

Ignacio Malagon

United Kingdom

Andrea Mangini

Italy

Peter Manning

United States of America

Hua Mao

United States of America

Silvana Marasco

Australia

Ronan Margey

United States of America

Christophe Mariette

France

Antonino GM Marullo

Italy

Parwis Massoudy

Germany

Shoko Matsui

Japan

Kozo Matsuo

Japan

Katsunari Matsuoka

Japan

Kaoru Matsuura

Japan

Peter Matt

Switzerland

David McIlroy

Australia

Fritz Mellert

Germany

Krassimir Metodiev

Bulgaria

Antonio Miceli

Italy

Slobodan Micovic

Serbia

Ashley M. Miller

American Samoa

Daniel L Miller

United States of America

Douglas Minnich

United States of America

Karl Mischke

Germany

Veselin Mitrovic

Germany

Akira Mogi

Japan

Fernando Moraes

Brazil

Luiz Felipe Moreira

Brazil

Thomas Muley

Germany

Leonardo Mulinari

Brazil

Mingyon Mun

Japan

Ashok Muniappan

United States of America

Henrique Murad

Brazil

Shuji Murakami

Japan

Bruno Murzi

Italy

Giuseppe Muscolino

Italy

Patrick Myers

United States of America

Mahdi Najafi

Iran

Kozo Nakanishi

Japan

Toshihiro Nanki

Japan

Giovanni Nano

Italy

Giuseppe Nasso

Italy

Mridu Paban Nath

India

Ehsan Natour

Netherlands

Saman Nazarian

United States of America

Petr Nemec

Czech Republic

Claus Nill

Germany

Vinicius Nina

Brazil

Nicolas Noiseux

Canada

Takashi Nojiri

Japan

Hiroaki Nomori

Japan

Tugrul Norgaz

Turkey

Luc Noyez

Netherlands

Martin Oberhoffer

Germany

Rupert Oberhuber

Austria

Stacy O'Blenes

Canada

Mehmet Oc

Turkey

Takeshi Oda

Japan

Vahagn Ohanyan

United States of America

Toshiya Ohtsuka

Japan

Jiro Okami

Japan

Homare Okamura

United States of America

Ken Olaussen

France

Wu Om

Japan

Masamichi Ono

Germany

Sedat Ozcan

Turkey

Hakan Ozhan

Turkey

Kanat Ozisik

Turkey

Emre Özker

Turkey

Mehmet O¿uzhan Özyurtkan

Turkey

Sanjay Pai

India

Chul-Hyun Park

South Korea

Kisung Park

South Korea

Seong Yong Park

South Korea

Himanshu Patel

United States of America

Marios Patronis

United Kingdom

Stefano Pelenghi

Italy

David Perez

Spain

Sossio Perrotta

Sweden

Predrag Perunovic

Serbia

Renzo Pessotto

United Kingdom

Francesco Petrella

Italy

Orlando Petrucci

Brazil

Hans K Pilegaard

Denmark

Francesco Pizzuto

Italy

Marek Pojar

Czech Republic

Eugenio Pompeo

Italy

Alain Poncelet

Belgium

Aron Frederik Popov

Germany

Italo Porto

Italy

Brian Prendergast

United Kingdom

Aristotelis Protopapas

United Kingdom

Ehud Raanani

Israel

Peter Raivio

Finland

Shahzad Raja

United Kingdom

Thomas Randsoe

Denmark

Vivek Rao

Canada

Giovanni Rapuzzi

Italy

Moheb Rashid

Sweden

Sudhir Rathore

United Kingdom

Mohamed Regal

Saudi Arabia

Syed Rehman

United Kingdom

Dinis Reis Miranda

Netherlands

Oliver Reuthebuch

Switzerland

Helena Rexius

Sweden

Mark Reynolds

United States of America

Gustavo Ribeiro

Brazil

Zaccaria Ricci

Italy

Michael Rigby

United Kingdom

Rezzani Rita

Italy

Gaetano Rocco

Italy

Marc-Estienne Roehrich

Argentina

Steven Rothenberg

United States of America

Abraham Rothman

United States of America

Nobhojit Roy

India

George Rozanski

United States of America

Enrico Ruffini

Italy

Claudio Francesco Russo

Italy

Bartosz Rylski

Poland

Han Young Ryu

South Korea

Eduardo Saadi

Brazil

Anton Sabashnikov

Germany

Mustafa Sacar

Turkey

Diyar Saeed

Germany

Francesco Saia

Italy

Francisco Saitua

Chile

Mohammad Sajjad

Saudi Arabia

Timothy Sakellaridis

Greece

Michele Salati

Italy

Andrea Salica

Italy

Cleusa Santos

Brazil

Carlos Santos-Gallego

United States of America

Motohiro Sato

Japan

Noriyoshi Sawabata

Japan

Shigeki Sawada

Japan

Thomas Scheeren

Netherlands

Benjamin Scherlag

United States of America

Jens-Christian Schewe

Germany

Orazio Schillaci

Italy

Christian Schreiber

Germany

Costas Schulze

Canada

Gerhard Schymik

Germany

Marcio Scorsin

Italy

Amir Sepehripour

United Kingdom

Dipesh Shah

India

Mahesh Sharma

United States of America

Shahriar Shayan

United States of America

Tomoki Shimokawa

Japan

Yanning Shou

United States of America

Andrea Siani

Italy

Alan Sihoe

Hong Kong

Brendan Silbert

Australia

Martin Simek

Czech Republic

Prasanna Simha M

India

Stavros Siminelakis

Greece

Mukesh Singh

United States of America

Horia Sirbu

Germany

Dirk Smets

Belgium

David Smith

United Kingdom

Ian Smith

United Kingdom

Luciano Solaini

Italy

Meong Gun Song

South Korea

Sandra Starnes

United States of America

Vaughn Starnes

United States of America

Sebastian Stec

Poland

Jie Sun

China

Ping Sun

United States of America

Xiumei Sun

United States of America

Michihiro Suwa

Japan

Imad Tabry

United States of America

Federico Tacconi

Italy

Shinzo Takamori

Japan

Hiroo Takayama

United States of America

Manabu Takebe

Japan

Koichi Takimoto

Japan

Masaya Tamura

Japan

Tawee Tanvetyanon

United States of America

Giordano Tasca

Italy

Carlo Tascini

Italy

Tolga Tatar

Turkey

Morteza Tavakkoli Hosseini

United Kingdom

Eiki Tayama

Japan

Deepak Tempe

India

Mark Tettey

Ghana

Oliver Michel Theusinger

Switzerland

Jess Thompson

United States of America

Hans Gustav Thyregod

Denmark

Mehmet Topçuoğlu

Turkey

Tiziano Torre

Switzerland

Juan A. Tovar

Spain

Flora Hau Fung Tsang

Hong Kong

Victor Tsang

United Kingdom

Joseph Turek

United States of America

Marko Turina

Switzerland

James Tweddell

United States of America

Naomichi Uchida

Japan

Kazuhiro Ueda

Japan

Murat Ugurlucan

Turkey

Akif Undar

United States of America

Daniel Unic

Croatia

Hidetaka Uramoto

Japan

Akihiko Usui

United Kingdom

Miguel Uva

Portugal

Orhan Uzun

United Kingdom

Vittorio Vanini

Italy

Rajamiyer Venkateswaran

United Kingdom

Prem Venugopal

United Kingdom

Murali Vettath

India

Jean-Louis Vincent

Belgium

Taisia Vitkovski

United States of America

Hunaid Vohra

United Kingdom

Marcel Vollroth

Germany

Song Wan

China

Weipeng Wang

China

Yigang Wang

United States of America

Zhiquan Wang

United States of America

Zhonggao Wang

China

Yuko Waseda

Japan

Atsushi Watanabe

Japan

Douglas West

United Kingdom

Grayson Wheatley

United States of America

Glenn Whitman

United States of America

Christoph Wiese

Germany

Antony Workman

United Kingdom

Qingyu Wu

China

Yu-Chung Wu

Taiwan

Harry Xia

United States of America

Naoya Yamasaki

Japan

Haydar Yasa

Turkey

Ali Yildiz

Turkey

Hiroyasu Yokomise

Japan

Nizar Yonan

United Kingdom

Junji Yoshida

Japan

Yoshio Yoshida

Japan

Hiroyoshi Yoshinami

Ireland

Lei Yu

China

Yang-Hao Yu

Taiwan

Yunfei Yuan

China

Louagie Yves

Belgium

Marina Zamith

Brazil

Qi Zeng

China

Qiu Zhibing

China

Claudio Zussa

Italy

